# Entlassmanagement in der Kinder- und Jugendpsychiatrie: Erwartungen und Realitäten aus Sicht der Eltern

**DOI:** 10.1007/s00115-020-00974-1

**Published:** 2020-08-11

**Authors:** I. Boege, B. Williams, U. Schulze, J. M. Fegert

**Affiliations:** 1 Weissenau, ZfP Südwürttemberg, Weingartshoferstraße 2, 88214 Ravensburg, Deutschland; 2 KJPP, Universität Ulm, Steinhövelstraße 5, 89075 Ulm, Deutschland; 3 Böblingen, KJPP, ZfP Nordschwarzwald, Bunsenstraße 120, 71032 Böblingen, Deutschland

**Keywords:** Versorgung, Schnittstellenmanagement, Koordination, Kinder- und Jugendliche, Psychische Erkrankung, Provision of care, Interface management, Coordination, Children and adolescents, Mental health

## Abstract

**Hintergrund und Fragestellung:**

Schnittstellen zwischen stationärer Behandlung psychisch erkrankter Kinder und Jugendlicher in der Kinder- und Jugendpsychiatrie, Jugendhilfe, Schulen, Arbeitsamt und Justiz sind nachweislich Sollbruchstellen für eine gelingende Schnittstellenkoordination im Sinne eines Entlassmanagements. Kriterien für ein Entlassmanagement in der Kinder- und Jugendpsychiatrie existieren nicht. Ziel der Studie ASpeKT (Aussagen zu Schnittstellenkoordination bei psychisch erkrankten Kindern und Teens) war es, den Iststand von Schnittstellenmanagement anhand von Aussagen von Eltern zu erheben und Empfehlungen für ein Entlassmanagement abzuleiten.

**Methoden:**

Zu zwei Zeitpunkten (T3 = 6 Monate, T4 = 12 Monate nach Entlassung) wurden Eltern (T3: *n* = 124, T4: *n* = 81) zu den erfolgten Hilfen und deren Koordination befragt.

**Ergebnisse:**

Eltern benennen, dass erreichbare poststationäre Hilfen für eine Stabilität nach stationärer Behandlung essenziell sind und einer guten Koordination bedürfen. Für eine gelingende Schnittstellenkoordination werden aus Sicht der Eltern benannt: vorhandene Case-Manager, frühzeitige Runde Tische, eine gute Übergabe an schulische Strukturen, nahtlose Anschlusstermine zur ambulanten Weiterbehandlung sowie Information zu Anlaufstellen und Behandlungsmöglichkeiten.

**Schlussfolgerung:**

Proaktive frühzeitige individuelle Koordination von Hilfen durch einen konstanten Ansprechpartner ist aus Sicht der betroffenen Familien für ein gutes Entlassmanagement essenziell.

## Abstract (en)

**Background:**

Interface management after inpatient care for mentally ill children and adolescents has been proven to be a breaking point in good transition of care between child and adolescent psychiatry, social welfare services, schools, job centre and the judicial system. Criteria for successful discharge management do not exist in child and adolescent psychiatry. Aim of the study ASpeKT was to survey parents on their perception of interface management and to derive recommendations for discharge management.

**Methods:**

Data regarding interface management were retrieved from parents (T3, *n* = 124, T4, *n* = 81) 6 months (T3) and 12 months (T4) after discharge.

**Results:**

The parents stated that accessible help after discharge from inpatient treatment is essential for stability and requires a good coordination. Parents named that they perceived helpful for successful interface management: a case manager, early round table meetings, support in returning to school, seamless access to outpatient follow-up appointments as well as information on further treatment options and contact data.

**Conclusion:**

From the perspective of affected families a proactive early individual and reliable care coordination by a constant contact person is essential for a good discharge management.

Der Rahmenvertrag Entlassmanagement (§ 39 Abs. 1a Sozialgesetzbuch V [SGB V]), welcher den Übergang von stationärer Krankenhausbehandlung zu ambulanten Hilfen regelt, trat verpflichtend zum 01.07.2017 in Kraft. Bis heute existieren aber keine klaren evidenzgestützten Empfehlungen für ein Entlassmanagement bei Kindern- und Jugendlichen mit psychischen Erkrankungen. Die Studie ASpeKT (Aussagen zu Schnittstellenkoordination bei psychisch erkrankten Kindern und Teens) hat anhand subjektiver Aussagen der Eltern den Iststand des derzeitigen Schnittstellenmanagements erfasst. Empfehlungen für eine gelingende Schnittstellenkoordination im Sinne des Entlassmanagements bei psychisch erkrankten Kindern und Jugendlichen werden abgeleitet.

Psychische Störungen bei Kindern und Jugendlichen sind mit 10–20 % häufig [1, 16] und gehen mit erheblichen psychosozialen Beeinträchtigungen im Alltag einher. Einerseits sind differenzierte psychiatrisch/psychotherapeutische Hilfen von niederschwelligen ambulanten Angeboten bis hin zu intensiven stationären Strukturen von Nöten [5]. Andererseits bedarf es bei diesen Patienten parallel bestehender psychosozialer Angebote der Jugendhilfe nach SGB VIII [4], Hilfen für Menschen mit Behinderungen (z. B. mit Autismusspektrumstörungen) nach SGB XI, Hilfen der schulpsychologischen Beratung, Schulbegleiter, Schulsozialarbeit inklusive Beschulung [6, 19], Erziehungsberatung, Hilfen des Arbeitsamtes, Kooperationen mit der Justiz sowie der Zusammenarbeit mit der Pädiatrie und Erwachsenenpsychiatrie.

Obwohl die Relevanz der Kooperation zwischen Kinder- und Jugendpsychiatrie und Strukturen außerhalb des SGB V seit langem bekannt ist [4], kommt es aber oftmals genau an dieser Sollbruchstelle zu Schwierigkeiten in der gelingenden Versorgung psychisch kranker Kinder und Jugendlicher. Spezifische Rollen einzelner Fachdisziplinen, Aufgaben und Strukturen der jeweiligen Systeme prallen aufeinander [20] und erschweren das nahtlose Ineinandergreifen von Hilfen. Besser integrierte Hilfen müssen hier aber das Ziel sein, damit eine effizientere Nutzung vorhandener Strukturen erfolgen kann.

Krankenhäuser sind inzwischen nach § 39 Absatz 1a SGB V [8] dazu verpflichtet, ein effektives Entlassmanagement zur Unterstützung des Übergangs in die Anschlussversorgung zu gewährleisten. Mit dem Gesetz zur Stärkung der Versorgung in der gesetzlichen Krankenversicherung (GKV-Versorgungsstärkungsgesetz – GKV-VSG, 2015) wurde das Entlassmanagement umfassend reformiert. Der Rahmenvertrag Entlassmanagement trat am 01.07.2017 verbindlich in Kraft. Dennoch fehlen klare Empfehlungen, wie ein Entlassmanagement an den Schnittstellen der Kinder- und Jugendpsychiatrie aussehen kann, welches nicht primär Hilfen des SGB V als Anschlussversorgung nutzt. Aus professioneller Sicht sind die skizzierten Schwierigkeiten bekannt [17, 19, 20], der Istzustand aus Sicht der Patienten und deren Eltern wurde bisher noch nicht erhoben.

Die Studie ASpeKT (**A**ussagen zu **S**chnittstellenkoordination bei **p**sychisch **e**rkrankten **K**indern und **T**eens)^1^ wollte nun diese Lücke schließen und anhand einer beobachtenden Befragung von Eltern 6 Monate und 12 Monate nach Entlassung aus stationärer Behandlung einen Iststand zu dem erfolgten Schnittstellenmanagement zwischen Hilfen des SGB V, SGB VIII, SGB XI, des Schulsystems, des Arbeitsamtes und der Justiz erheben. Empfehlungen für das gesetzlich vorgegebene Entlassmanagement sollten aus den Aussagen der Eltern abgeleitet werden.

## Methode

### Stichprobe

Die qualitative Datenerhebung der ASpeKT-Studie fand zwischen dem 22.04.2015 und dem 22.03.2018 an zwei Standorten statt. Ein standardisiertes Entlassmanagement erfolgte an keinem der beiden Standorte während des Verlaufs der Studie. Allen stationär aufgenommenen Patienten im Alter von 5 bis 18 Jahren, welche die Einschlusskriterien erfüllten (Indikation zur stationären Behandlung aufgrund einer diagnostizierbaren psychiatrischen Störung; Verbleib auf der Station >24 h; IQ > 69, da qualitativen Aussagen von Eltern und Kindern/Jugendlichen erhoben werden sollten; Beherrschen der deutschen Sprache) wurde eine Beteiligung an der Studie angeboten.

Standort 1 versorgt dabei mit 30 vollstationären Betten ca. eine Einwohnerzahl von 600.000 Menschen. Standort 2 versorgt ca. eine Einwohnerzahl von 500.000 Menschen mit insgesamt 27 vollstationären Betten. Erfolgte eine Einwilligung zur Teilnahme an der Studie (Eltern und Patienten) im Sinne des „informed consent“ [15] fand innerhalb von 2 Wochen nach Aufnahme (T1), zum Zeitpunkt der Entlassung (T2), nach 6 Monaten (T3) und nach 12 Monaten (T4) einerseits eine quantitative Datenerhebung [2] und andererseits eine qualitative telefonische Befragung der Eltern durch einen von zwei wissenschaftlichen Mitarbeitern statt.

Bei T1 fand eine persönliche Befragung der Kinder- und Jugendlichen statt, während eine Kontaktaufnahme zu den Eltern telefonisch stattfand. In den folgenden Interviews zu T2, T3 und T4 wurde generell eine telefonische Befragung anstelle des Face-to-face-Interviews gewählt, da neben ökonomischen Vorteilen eine höhere Ausschöpfungsquote erwartet wurde [3]. Vogl [18] stellte dar, dass auch für Kinder halbstrukturierte Telefoninterviews gut geeignet sind, insbesondere wenn ein vorheriger Face-to-face-Kontakt stattgefunden habe. Hier ausgewertet wurden die qualitativen Aussagen der Eltern zum Zeitpunkt T3 und T4.

### Datenerhebung und Instrument

Die Datenerhebung erfolgte zu den Zeitpunkten T3 und T4 anhand eines halbstandardisierten Interviewleitfadens. Dieser wurden von einem Expertenteam (drei Fachärzte/innen für Kinder- und Jugendpsychiatrie und Psychotherapie, zwei Psychologinnen) entwickelt. Die Interviews wurden parallel in den dafür vorgesehenen Bögen aufgezeichnet und im Anschluss mithilfe von Word und Excel verarbeitet (Tab. 1).

**Table 1:** 

Zeitpunkt	Fragestellungen
*T3, T4*	Welche Hilfen haben Sie/Ihr Kind in den letzten 6 bzw. 12 Monaten erhalten?
	Haben diese etwas für Ihre Familie verändert?
	Wenn Sie und Ihr Kind mehr als eine Hilfeform erhalten haben, gab es eine Kommunikation und Koordination zwischen den Hilfssystemen?
	Haben Sie auf Hilfen warten müssen?
	Haben Sie sich, als ihr Kind aus der stationären Behandlung entlassen wurde, auch in der Übergangszeit gut betreut gefühlt?
	Haben sich Übergänge problemlos gestaltet, sodass sich keine Lücken im Erhalt von Hilfen ergeben haben?
	Was hätte besser laufen können?

T3 = 6 Monate nach Entlassung, T4 = 12 Monate nach Entlassung

Die Studie wurde mit Zustimmung der Ethikkommission der Universität Ulm im Einklang mit nationalem Recht sowie gemäß der Deklaration von Helsinki von 1975 (in der aktuellen, überarbeiteten Fassung) durchgeführt. Von allen Patienten und deren sorgeberechtigten Eltern liegt eine schriftliche Einverständniserklärung vor.

### Datenanalyse

Die Datenanalyse erfolgte nach der Methode der qualitativen Inhaltsanalyse von Mayring [13]. Die Interviews dauerten zwischen 5 und 25 min und wurden wörtlich aufgezeichnet. Mehrfachnennungen waren möglich. Eine einzelne Kodierung erfolgte, wenn verschiedene Aspekte abgebildet wurden.

Während der laufenden Datenerhebung wurden Kategorien schrittweise induktiv aus der Dokumentation der Telefoninterviews extrahiert. Die Kategorien wurden nach Kodierung von 50 % des Materials in einer Konsensuskonferenz diskutiert, das Kategoriensystem revidiert und die Telefoninterviews endgültig kodiert. Für jede Frage wurden zwischen 8 und 16 Kategorien gebildet.

Ankerbeispiele dienten der Operationalisierung und trugen dazu bei, die Reliabilität des Kategoriensystems zu gewährleisten. Insgesamt 20 % des Materials wurde zufällig ausgewählt und abgleichend von zwei unabhängigen Ratern kodiert. Die Interrater-Reliabilität wurde mit dem κ‑Koeffizient (Cohen 1960) berechnet [14]. Die Interrater-Reliabilität betrug durchschnittlich κ = 0,83, was nach Landis und Koch [12] als sehr hohe Übereinstimmung zu werten ist.

### Statistische Auswertung

Die statistische Auswertung erfolgte mit IBM SPSS 25.0. Deskriptive Statistiken dienen zur Beschreibung der Stichprobe. Mithilfe von t‑Tests für kontinuierliche Variablen und χ^2^-Tests für kategoriale Variablen wurde auf Unterschiede zwischen den Studiengruppen getestet (*α* = 0,05).

## Ergebnisse

### Stichprobe

**Figure 1:**
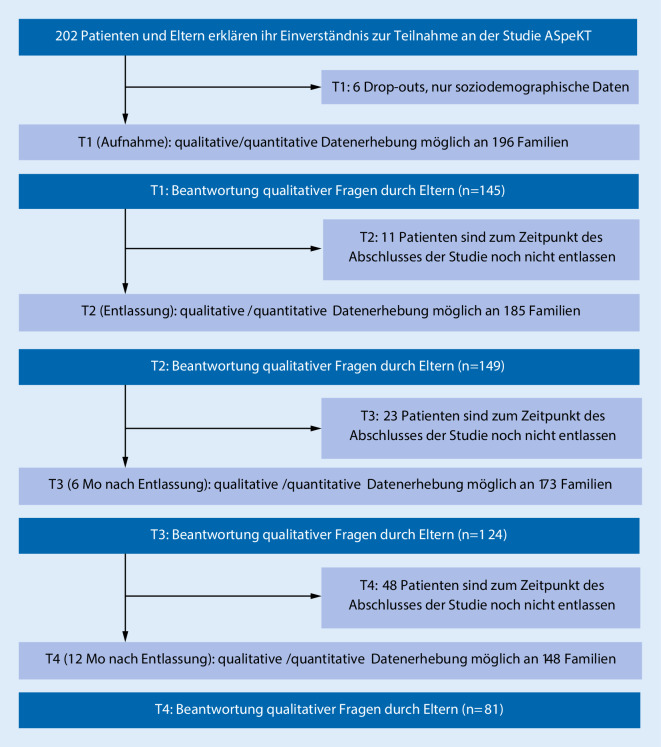

Insgesamt 202 Patienten und deren Eltern willigten ein, an der Studie ASpeKT teilzunehmen, bei 6 Fällen kam es direkt zu einem Drop-out. Für diese Fälle konnten nur soziodemografische Daten erhoben werden. Von den möglichen 196 Eltern erklärten sich 145 Eltern bereit zusätzlich zu den quantitativen Fragebögen auch qualitative Fragen zu beantworten. Zu T2 waren 149 Eltern bereit quantitative Fragen zu beantworten, zu T3 124 und zu T4 81. (Abb. 1).

Das Geschlechterverhältnis war ausgewogen (weiblich 52,5 % vs. männlich 47,5 %), es nahmen etwas weniger Eltern von Kindern (46,35 %) als von Jugendlichen (53,65 %) teil. Internalisierende Störungen kamen etwas häufiger vor (53,7 %) als externalisierende Störungen (40,8 %; vgl. Tab. 2).

**Table 2:** 

	Eingeschlossene Patienten (*n* = 202)
**Alter**	
*Kinder 5–13 Jahre*	46,35 %
*Jugendliche 14–17 Jahre*	53,65 %
**Geschlecht**	
*Weiblich*	52,5 %
*Männlich*	47,5 %
**Diagnose (ICD-10)**	
*Organische Störung (F0)*	0,6 %
*Psychotische Störung (F2)*	1,6 %
*Affektive Störung (F3)*	24,5 %
*Angst/Zwang/PTBS (F4)*	20,7 %
*Essstörung (F5)*	8,5 %
*Persönlichkeitsstörung (F6)*	0,6 %
*Entwicklungsstörung (F8)*	3,1 %
*Verhaltensstörung/ADHS (F9)*	40,8 %
Verweildauer Ø	67,9 Tage

### Welche Hilfen haben Sie/Ihr Kind in den letzten 6 bzw. 12 Monaten erhalten?

Am häufigsten benannten die befragten Eltern zum Zeitpunkt T3, dass ihr Kind noch eine ambulante Psychotherapie wahrnehme (*n* = 95). Zum Zeitpunkt T4 benannten dies noch 45 Eltern. Ebenfalls wurde von vielen Eltern benannt, dass ihr Kind Jugendhilfe erhalte bzw. sie mit dem Jugendamt im Kontakt sind (T3 = 64, T4 = 49). Kinder/Jugendliche hatten laut 36 Eltern zu T3 noch einen speziellen Ansprechpartner in der Schule, zum Zeitpunkt T4 waren das noch 19 der Kinder/Jugendlichen.

### Haben die erhaltenen Hilfen etwas für Ihre Familie verändert?

Zu beiden Zeitpunkten benannten die Eltern einen deutlichen Kompetenzgewinn des Kindes/Jugendlichen (T3 *n* = 50, T4 *n* = 57; z. B.: „Jetzt kann sie ihre Gefühle verarbeiten, ist authentischer“, „Sie hat gelernt Skills einzusetzen.“) Wenige Eltern benannten einen Kompetenzzugewinn bei sich selbst: (z. B. *„*Ich sah klarer die Situation und lernte Grenzen abzustecken“; T3 *n* = 7, T4 *n* = 11). In beiden Gruppen kam es zu T3 und T4 zu einer persönlichen Entlastung der Eltern (T3 *n* = 30, T4 *n* = 24; Tab. 3).

**Table 3:** 

	T3 (6 Mo)	T4 (12 Mo)
Kompetenzzugewinn Kind	50 (40 %)	57 (70 %)
Kompetenzzugewinn Eltern	7 (7 %)	11 (14 %)
Kommunikation in der Familie (nur Eltern)	16 (13 %)	7 (9 %)
Entlastung Eltern/Entlastung Familie	30 (24 %)	24 (30 %)
Negative Entwicklung	14 (11 %)	14 (17 %)
Settingwechsel	12 (10 %)	19 (23 %)
Gleich geblieben	9 (7 %)	8 (10 %)

### Übergänge und Koordination von Hilfen

Eltern gaben zu T3 und zu T4 an, dass die installierten Hilfssysteme in fast der Hälfte der Fälle nicht miteinander im Kontakt waren (z. B.: „Eine Koordination von außen gab es nicht“, T3 *n* = 42, T4* n* = 35). Zum Zeitpunkt T3 gaben 59 der Eltern an, auf Hilfen für ihr Kind gewartet zu haben, zu T4 war dies noch bei 12 der Fall. An den Übergängen (T3) fühlten sich 51 der befragten Eltern gut begleitet, auch zum Zeitpunkt T4 fühlten sich viele Eltern (*n* = 29) noch gut an Übergängen begleitet (z. B.: „Unterstützungssysteme waren und sind für das Kind da.“). Zum Zeitpunkt T3 gab die Hälfte der Eltern (*n* = 54) an, durchgängig Hilfen erhalten zu haben, zum Zeitpunkt T4 sind es noch 33 (z. B. „Wir konnten uns jederzeit an den Arzt, seine Betreuer, den Sozialarbeiter wenden/diesen anrufen.“).

### Was hätte besser laufen können?

Eine bessere Organisation der Nachsorge/Schnittstellen hätten sich 61 Eltern zu T3 gewünscht, zu T4 waren es noch 52 Eltern („Wir mussten uns um alles selbst kümmern.“, „…dass wir eine Koordination im Übergang erfahren hätten“). Das Familiensystem mehr im Blick zu haben, wäre 44 Eltern zu T3 und 20 Eltern zu T4 ein Anliegen gewesen (z. B. *„*Ich hätte mir gewünscht, einbezogen zu werden und mitzulernen“; Tab. 4).

**Table 4:** 

	T3 (6 Mo)	T4 (12 Mo)
Bessere Organisation der Nachsorge/Schnittstellen	61 (49 %)	52 (64 %)
Übergang Schule	18 (14 %)	11 (13 %)
Bessere Passung: Therapeut – Kind/Jugendlicher	18 (14 %)	7 (9 %)
Familiensystem besser im Blick haben	44 (35 %)	20 (25 %)
Nachhaltiger Therapieerfolg	9 (7 %)	6 (7 %)
Sonstige	8 (6 %)	5 (6 %)
Nichts	18 (14 %)	20 (25 %)

## Diskussion

Ziel der Studie ASpeKT war es, den Iststand des subjektiven Erlebens von Eltern sowie Kindern/Jugendlichen an dem Übergang stationär/ambulant bzw. den Schnittstellen zwischen den Systemen nach einer stationären Behandlung zu erheben. Dies erfolgte zum Zeitpunkt T3 an 124 (61, 4 %) und zum Zeitpunkt T4 an 81 (40 %) Eltern der Originalstichprobe. Mittels der vorliegenden Aussagen der Eltern sollten vorsichtig praktische Empfehlungen für ein tragendes Schnittstellenmanagement abgeleitet werden. Vorgabe der Gesetzgebung ist, dass zur „Gewährleistung eines nahtlosen Übergangs der Patienten in die nachfolgenden Versorgungsbereiche ein Krankenhaus durch die Anwendung eines geeigneten Assessments den patientenindividuellen Bedarf für die Anschlussversorgung möglichst frühzeitig erfassen und ein Entlassplan aufstellen soll“ [9]. Gerade in der Kinder- und Jugendpsychiatrie ist es für das Entlassmanagement von hoher Relevanz, nicht nur die psychotherapeutische Nachsorge nach SGB V im Blick zu haben, sondern – wo erforderlich – frühzeitig Vertreter der Schule, Jugendhilfe, Arbeitsamt, Justiz sowie Kollegen der Pädiatrie und Erwachsenenpsychiatrie mit einzubeziehen. Die Komplexität der Symptomatik ist bei Kindern und Jugendlichen mit psychischen Störungen nur interdisziplinär verstehbar und zu bewältigen.

Eltern benannten in den Interviews, dass ein koordinierender Hilfeansprechpartner hilfreich wäre: mit rechtlicher Expertise, Schnittstelleninformation, Koordinationsmöglichkeiten und Verständnis für die individuelle familiäre Belastung. Explizit wird zu beiden Zeitpunkten von vielen Eltern eine bessere Organisation der Nachsorge, die das Familiensystem als Ganzes im Blick hat, benannt. Bei längeren Verläufen nehmen eigene Wünsche der Eltern nach Psychoedukation und Coaching im Umgang mit ihrem Kind zu. Ebenfalls benannten die Eltern Präsenz und Beziehung zum Therapeuten als wichtig. Abzuleiten ist aus den Aussagen der Eltern: **Empfehlung 1:** Kinder/Jugendliche mit einer stationär behandlungsbedürftigen psychischen Erkrankung sollten einen Case-Koordinator mit einem Stundenkontingent erhalten, der im Sinne von Einzelfall-Case-Management oder Hilfeplankonferenzen [21] als systemübergreifender Ansprechpartner für die Familie zuständig ist. Dieser sollte frühzeitig, noch während des stationären Aufenthalts, die Brücke nach Hause schlagen, Hilfen für den Zeitraum nach der Entlassung organisieren und auch im Nachgang des stationären Aufenthalts noch für die Familie als zentraler Ansprechpartner, auch bei Krisen, bestehen bleiben.

Ebenfalls wurde angegeben, dass die installierten Hilfssysteme in fast der Hälfte der Fälle nicht miteinander im Kontakt waren bzw. dass Eltern auf Hilfen für ihr Kind lange warten mussten (T3). **Empfehlung 2:** Bei absehbar komplexen Fällen sollte frühzeitig ein „round table“ (in den ersten zwei Wochen nach Aufnahme) durchgeführt werden. So bleibt genügend Zeit, um gemeinsam mit den möglichen Helfern die bestehende Symptomatik zu erfassen und gemeinsam Lösungsvorschläge aus verschiedenen Blickwinkeln zu entwickeln.

Dabei stehen jugendhilfliche und psychotherapeutische Maßnahmen nach einer stationären Aufnahme im Vordergrund. Jugendhilfe wurde zum Zeitpunkt T3 von 62 % (*n* = 78) und zum Zeitpunkt T4 von 55,5 % (*n* = 49) der Patienten wahrgenommen, ambulante Psychotherapie zum Zeitpunkt T3 von 63,3 % (*n* = 81) und zum Zeitpunkt T4 von 66,4 % (*n* = 58). Dies zeigt deutlich den Bedarf einer nachstationär erreichbaren und vor allem nahtlosen ambulanten Weiterbehandlung. Nur wenige Patienten gaben an, keine Nachsorge mehr wahrzunehmen. Eltern der ASpeKT-Studie beklagten zum Teil, dass sie auf Nachsorge lange hätten warten müssen. Gleichzeitig wurde übereinstimmend mit den Ergebnissen in der KIGGS-Studie [7, 16] benannt, dass lange Anfahrtswege das Wahrnehmen einer nötigen Psychotherapie erschweren. **Empfehlung 3:** Eine flächendeckende Therapeutendichte ist sicherzustellen, um gerade ländliche Regionen gut anzubinden.

Nicht nur die therapeutischen und jugendhilflichen Angebote bedürfen dabei Verzahnung, so wird von Eltern benannt, dass beim Übergang von der Klinik nach Hause bisher besonders der Wiedereinstieg in die Heimatschule nicht gut vorbereitet gewesen sei. Sie hätten sich hier mehr Begleitung gewünscht, damit das Kind/der Jugendliche nicht an dem wieder einsetzenden Anforderungsniveau der Schule scheitern würde. **Empfehlung 4:** Einbezug der Schule im Übergang, z. B. durch Beteiligung der Heimatschule an einem „round table“ vor der Entlassung. Alternativ wäre ein regelhaftes Angebot aufsuchender Psychoedukation der Lehrer bzw. Verweis auf den den Kindern und Jugendlichen zustehenden Nachteilsausgleich § 3 Abs. 5 Schulgesetz [10, 11] als Teil des Entlassmanagements zu verstehen.

Aus einigen Aussagen der Eltern wird deutlich, dass die Eltern sich bei der Suche nach Hilfen nicht immer ernst genommen gefühlt haben oder aber basisnahe Informationen, wie z. B. Adresslisten von Angeboten, schwer zu erhalten gewesen seien. **Empfehlung 5:** Erstellen einer systemübergreifenden Informationsbroschüre für alle Helfersysteme der Region (Medizin, Jugendamt, Schule, Arbeitsamt, Justiz) mit Adressen, Erreichbarkeiten und dem jeweiligen Angebot der vorgehaltenen Hilfen. Diese Broschüre sollte den Eltern und Kindern/Jugendlichen bei einem Ersttermin zugänglich gemacht werden, unabhängig von der Instanz, bei der zuerst Hilfe gesucht wird. So werden Eltern als Experten für ihr Kind wahrgenommen und zu gleichberechtigten Partnern bei der Wahl der Hilfen für ihr Kind gemacht.

Limitation der Studie ist, dass es aufgrund des vorbestimmten Endzeitpunkts und einer zu Beginn langsam anlaufenden Patientenrekrutierung zu einer Reduktion der zu den Zeitpunkten T3 und T4 zu befragenden Familien kam. Zudem zeigte sich zum Zeitpunkt T4 ein relativ hoher Drop-out von 49 %. Eine weitere Einschränkung in der Interpretation der Ergebnisse ergab sich aus der Tatsache, dass eine Einwilligung von Patienten zur Teilnahme an der Studie eher von Patienten und deren Eltern erfolgte, die eine längere Verweildauer aufwiesen. Die Ergebnisse der Studie sind so eher für Patienten, welche eine Regelbehandlung wahrnehmen, als gültig anzusehen. Zuletzt ist zu hinterfragen, ob alle Hilfsvorschläge, die seitens der Therapeuten bzw. des Teams gemacht wurden, von den Eltern beachtet bzw. aufgegriffen wurden. Oder ob nicht auch manche nicht umgesetzt wurden, dann aber zum Zeitpunkt der Befragung, entweder als Defizit in der Versorgung gewertet bzw. nicht erinnert wurden.

## Fazit für die Praxis

- Kinder und Jugendliche mit psychischen Störungen benötigen zum Zeitpunkt der Entlassung aus der stationären Behandlung ein gutes Ineinandergreifen der verschiedenen Systeme unter Einbezug des Familiensystems. Der Bedarf von Anschlussmaßnahmen in den Systemen Psychotherapie, Jugendamt, Schule ist hoch. Eine Informationsbroschüre mit systemübergreifenden Hilfsangeboten, Telefonnummern und Ansprechpartner pro Region sollte Eltern, Kindern und Jugendlichen frühzeitig zur Verfügung gestellt werden.
- Es bedarf während des stationären Aufenthalts einer proaktiven individuellen frühzeitigen Koordination der möglichen nötigen Hilfen. Runde Tische mit Klärung der anzugehenden Problembereiche außerhalb der Klinik, sollten möglichst schon bei Aufnahme auf die Station erfolgen.
- Schulen sollten in den Entlassprozess mit einbezogen werden, um ggf. schulische Anforderungen den Möglichkeiten des Kindes/der Jugendlichen zeitbefristet anzupassen. Dadurch wäre den Kindern/Jugendlichen mit psychischen Störungen die Reintegration in die Schule zu erleichtern.

## Footnotes

1 Von der Stiftung Kinderland, Baden-Württemberg, gefördert.
